# 5-*tert*-Butyl-2-hy­droxy-3-(2-thien­yl)benzaldehyde

**DOI:** 10.1107/S1600536810030382

**Published:** 2010-08-04

**Authors:** Yanwei Wang, Zhenzhen Qiu, Hongze Liang

**Affiliations:** a Faculty of Materials Science and Chemical Engineering, Ningbo University, Ningbo, Zhejiang 315211, People’s Republic of China

## Abstract

In the crystal structure of the title compound, C_15_H_16_O_2_S, the thio­phene ring is essentially planar (r.m.s. deviation = 0.006 Å for all non-H atoms) and roughly coplanar with the benzene ring, the dihedral angle between the mean planes of the rings being 4.35 (8)°. An intra­molecular O—H⋯O hydrogen bond is observed between the OH group and the aldehyde O atom.

## Related literature

For related salicyl­aldehyde derivative compounds, see: Qiu *et al.* (2009 ▶); Yu *et al.* (2007 ▶); Wang *et al.* (2009 ▶); Wong *et al.* (2004 ▶). 
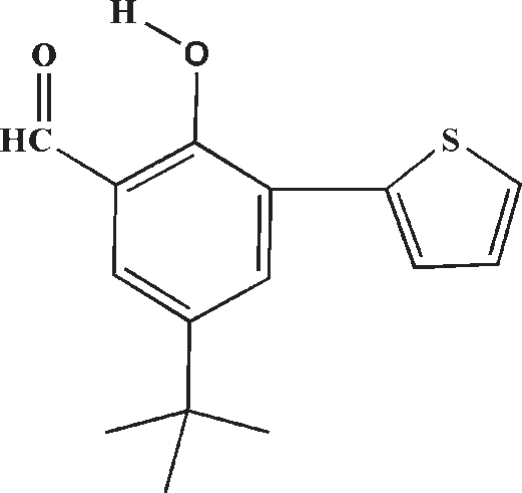


## Experimental

### 

#### Crystal data

C_15_H_16_O_2_S
*M* *_r_* = 260.34
Triclinic, 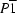

*a* = 7.2016 (14) Å
*b* = 8.9375 (18) Å
*c* = 10.922 (2) Å
α = 91.50 (3)°
β = 107.69 (3)°
γ = 93.25 (3)°
*V* = 668.0 (2) Å^3^
*Z* = 2
Mo *K*α radiation
μ = 0.23 mm^−1^
*T* = 173 K
0.10 × 0.10 × 0.10 mm

#### Data collection

Rigaku R-AXIS RAPID diffractometer
Absorption correction: multi-scan (*ABSCOR*; Higashi, 1995 ▶) *T*_min_ = 0.977, *T*_max_ = 0.977
5891 measured reflections
2705 independent reflections
1958 reflections with *I* > 2σ(*I*)
*R*_int_ = 0.020

#### Refinement

*R*[*F* ^2^ > 2σ(*F* ^2^)] = 0.059
*wR*(*F* ^2^) = 0.208
*S* = 1.15
2705 reflections
179 parameters
9 restraints
H atoms treated by a mixture of independent and constrained refinement
Δρ_max_ = 0.41 e Å^−3^
Δρ_min_ = −0.45 e Å^−3^

### 

Data collection: *RAPID-AUTO* (Rigaku, 1998 ▶); cell refinement: *RAPID-AUTO*; data reduction: *CrystalStructure* (Rigaku/MSC, 2004 ▶); program(s) used to solve structure: *SHELXS97* (Sheldrick, 2008 ▶); program(s) used to refine structure: *SHELXL97* (Sheldrick, 2008 ▶); molecular graphics: *SHELXTL* (Sheldrick, 2008 ▶); software used to prepare material for publication: *SHELXL97*.

## Supplementary Material

Crystal structure: contains datablocks I, global. DOI: 10.1107/S1600536810030382/zq2050sup1.cif

Structure factors: contains datablocks I. DOI: 10.1107/S1600536810030382/zq2050Isup2.hkl

Additional supplementary materials:  crystallographic information; 3D view; checkCIF report

## Figures and Tables

**Table 1:** Hydrogen-bond geometry (Å, °)

*D*—H⋯*A*	*D*—H	H⋯*A*	*D*⋯*A*	*D*—H⋯*A*
O2—H2*D*⋯O1	0.78 (3)	1.89 (3)	2.623 (3)	157 (3)

**Fig. 1.**
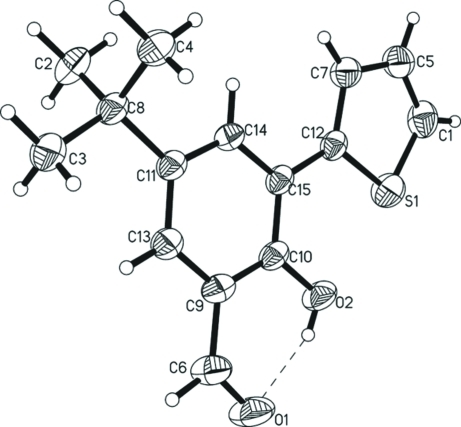
View of (I) with displacement ellipsoids drawn at the 30% probability level. The dotted line indicates the intramolecular H-bond.

## supplementary crystallographic information

### Comment

Salicylaldehyde and its derivatives are widely used in the construction of metal complexes (Qiu *et al.*, 2009; Wang *et al.*, 2009; Yu *et al.*, 2007). We have synthesized a series of lanthanide complexes of a Schiff base which derived from 2-pyridyl salicylaldehyde and investigated their luminescent properties (Wong *et al.*, 2004). In the course of exploring new luminescent compounds, we synthesized the title molecule 5-*tert*-butyl-2-hydroxy-3-thiophen-2-ylbenzaldehyde (I) as an intermediate compound.

The molecular structure of the title compound is shown in Fig. 1. The thiophene ring is essentially planar (rms deviation = 0.006Å for all non-H atoms) and roughly coplanar with the phenyl ring, making a dihedral angle between the mean planes of the rings of 4.35 (8)°. There are no intermolecular hydrogen bonds in the crystal structure but the intramolecular interaction O2—H2D···O1 between the hydroxy OH group and the aldehyde O atom is helpful to stabilize the molecular conformation.

### Experimental

3-Bromo-5-*tert*-butyl-2-hydroxybenzaldehyde (1.50 g, 5.86 mmol), 2-thienyl-tributyltin (2.40 g, 6.45 mmol), triphenyl phosphine (0.31 g, 0.38 mmol) and palladium dichloride (0.05 g, 0.28 mmol) were added to 20 ml THF in a round flask, and this mixture was refluxed with agitation for 24 h. Then, 20 ml toluene was added and the mixture was refluxed at 100°C for another 24 h. Finally, the reaction mixture was refluxed at 105°C after addition of 20 ml DMF for further 24 h. After evaporating the solvent, the residue was chromatographed on silica gel and a yellowish precipitate was produced. The precipitate was recrystallized from dichloromethane and yellow block-shaped crystals were obtained (0.62 g, 41%). ^1^H NMR (400 MHz, CDCl_3_): 11.73 (s, 1*H*), 9.93 (s, 1*H*), 7.91 (d, J = 2.0 Hz, 1*H*), 7.31 (dd, J = 1.2 Hz, J = 3.6 Hz,1*H*), 7.48 (d, J = 2.4 Hz, 1*H*), 7.37 (dd, J = 4.8 Hz, J = 0.8 Hz, 1*H*), 7.25 (s, 1*H*), 7.13 (dd, J = 3.6 Hz, J = 4.8 Hz, 1*H*), 1.37 (s, 9*H*). MS (EI, m/*z*): 260[*M*]+

### Refinement

The H atoms attached to C atoms were placed in calculated positions and treated using a riding-model approximation (with C–H = 0.95 Å and *U*_iso_(H) = 1.2*U*_eq_(C) for aromatic H atoms, and with C–H = 0.98 Å and *U*_iso_(H) = 1.5*U*_eq_(C) for the methyl H atoms). The important H atoms bonded to O2 and C6 were located in the difference Fourier map and were freely refined.

### Crystal data

**Table:**

C_15_H_16_O_2_S	*Z* = 2
*M**_r_* = 260.34	*F*(000) = 276
Triclinic, *P*1	*D*_x_ = 1.294 Mg m^−^^3^
Hall symbol: -P 1	Mo *K*α radiation, λ = 0.71073 Å
*a* = 7.2016 (14) Å	Cell parameters from 5891 reflections
*b* = 8.9375 (18) Å	θ = 3.0–26.4°
*c* = 10.922 (2) Å	µ = 0.23 mm^−^^1^
α = 91.50 (3)°	*T* = 173 K
β = 107.69 (3)°	Block, yellow
γ = 93.25 (3)°	0.10 × 0.10 × 0.10 mm
*V* = 668.0 (2) Å^3^	

### Data collection

**Table:**

Rigaku R-AXIS RAPID diffractometer	2705 independent reflections
Radiation source: fine-focus sealed tube	1958 reflections with *I* > 2σ(*I*)
graphite	*R*_int_ = 0.020
Detector resolution: 0 pixels mm^-1^	θ_max_ = 26.4°, θ_min_ = 3.0°
ω scans	*h* = −8→8
Absorption correction: multi-scan (*ABSCOR*; Higashi, 1995)	*k* = −11→11
*T*_min_ = 0.977, *T*_max_ = 0.977	*l* = −13→13
5891 measured reflections	

### Refinement

**Table:**

Refinement on *F*^2^	Primary atom site location: structure-invariant direct methods
Least-squares matrix: full	Secondary atom site location: difference Fourier map
*R*[*F*^2^ > 2σ(*F*^2^)] = 0.059	Hydrogen site location: inferred from neighbouring sites
*wR*(*F*^2^) = 0.208	H atoms treated by a mixture of independent and constrained refinement
*S* = 1.15	*w* = 1/[σ^2^(*F*_o_^2^) + (0.1265*P*)^2^ + 0.0566*P*] where *P* = (*F*_o_^2^ + 2*F*_c_^2^)/3
2705 reflections	(Δ/σ)_max_ < 0.001
179 parameters	Δρ_max_ = 0.41 e Å^−^^3^
9 restraints	Δρ_min_ = −0.45 e Å^−^^3^

### Special details

**Table:**

Geometry. All e.s.d.'s (except the e.s.d. in the dihedral angle between two l.s. planes) are estimated using the full covariance matrix. The cell e.s.d.'s are taken into account individually in the estimation of e.s.d.'s in distances, angles and torsion angles; correlations between e.s.d.'s in cell parameters are only used when they are defined by crystal symmetry. An approximate (isotropic) treatment of cell e.s.d.'s is used for estimating e.s.d.'s involving l.s. planes.
Refinement. Refinement of *F*^2^ against ALL reflections. The weighted *R*-factor *wR* and goodness of fit *S* are based on *F*^2^, conventional *R*-factors *R* are based on *F*, with *F* set to zero for negative *F*^2^. The threshold expression of *F*^2^ > σ(*F*^2^) is used only for calculating *R*-factors(gt) *etc*. and is not relevant to the choice of reflections for refinement. *R*-factors based on *F*^2^ are statistically about twice as large as those based on *F*, and *R*- factors based on ALL data will be even larger.

### Fractional atomic coordinates and isotropic or equivalent isotropic displacement parameters (Å^2^)

**Table:**

	*x*	*y*	*z*	*U*_iso_*/*U*_eq_	
S1	0.39900 (12)	0.44272 (9)	0.30990 (6)	0.0798 (4)	
O2	0.3088 (3)	0.6482 (2)	0.12395 (19)	0.0677 (5)	
C15	0.2702 (3)	0.3967 (2)	0.0406 (2)	0.0482 (5)	
C14	0.2152 (3)	0.3004 (2)	−0.0692 (2)	0.0496 (5)	
H14A	0.2226	0.1957	−0.0574	0.060*	
C13	0.1403 (3)	0.4992 (3)	−0.2105 (2)	0.0544 (6)	
H13A	0.0979	0.5352	−0.2949	0.065*	
C12	0.3408 (3)	0.3350 (3)	0.1694 (2)	0.0508 (5)	
C11	0.1504 (3)	0.3476 (2)	−0.1946 (2)	0.0497 (5)	
C10	0.2573 (3)	0.5507 (3)	0.0206 (2)	0.0507 (5)	
C9	0.1912 (3)	0.6013 (3)	−0.1048 (2)	0.0556 (6)	
C8	0.0927 (4)	0.2304 (3)	−0.3084 (2)	0.0564 (6)	
C7	0.3726 (4)	0.1835 (3)	0.1953 (2)	0.0636 (6)	
O1	0.2186 (4)	0.8609 (2)	−0.0407 (2)	0.0947 (7)	
C6	0.1769 (5)	0.7611 (3)	−0.1262 (3)	0.0748 (8)	
C5	0.4400 (4)	0.1649 (3)	0.3312 (3)	0.0721 (7)	
H5A	0.4688	0.0706	0.3682	0.087*	
C4	0.2655 (4)	0.1362 (3)	−0.3025 (2)	0.0716 (7)	
H4A	0.3763	0.2017	−0.3080	0.107*	
H4B	0.2283	0.0616	−0.3746	0.107*	
H4C	0.3026	0.0851	−0.2212	0.107*	
C3	0.0331 (5)	0.3042 (3)	−0.4383 (2)	0.0759 (8)	
H3A	0.1425	0.3700	−0.4456	0.114*	
H3B	−0.0795	0.3635	−0.4446	0.114*	
H3C	−0.0017	0.2265	−0.5079	0.114*	
C2	−0.0816 (4)	0.1276 (3)	−0.2998 (3)	0.0757 (8)	
H2A	−0.1191	0.0532	−0.3721	0.114*	
H2B	−0.1922	0.1881	−0.3032	0.114*	
H2C	−0.0443	0.0761	−0.2187	0.114*	
C1	0.4584 (4)	0.2933 (4)	0.4010 (3)	0.0765 (8)	
H7	0.354 (6)	0.109 (3)	0.140 (3)	0.127 (14)*	
H2	0.504 (4)	0.303 (4)	0.4875 (10)	0.082 (9)*	
H6A	0.135 (6)	0.804 (5)	−0.213 (4)	0.127 (14)*	
H2D	0.292 (4)	0.726 (4)	0.093 (3)	0.068 (9)*	

### Atomic displacement parameters (Å^2^)

**Table:**

	*U*^11^	*U*^22^	*U*^33^	*U*^12^	*U*^13^	*U*^23^
S1	0.1003 (6)	0.0763 (6)	0.0558 (5)	0.0081 (4)	0.0148 (4)	−0.0179 (3)
O2	0.0845 (13)	0.0497 (11)	0.0657 (11)	−0.0018 (9)	0.0210 (10)	−0.0186 (9)
C15	0.0443 (11)	0.0505 (12)	0.0496 (11)	−0.0004 (9)	0.0157 (9)	−0.0095 (9)
C14	0.0550 (12)	0.0418 (11)	0.0503 (12)	0.0017 (9)	0.0146 (10)	−0.0067 (9)
C13	0.0561 (13)	0.0525 (13)	0.0551 (12)	0.0022 (10)	0.0183 (10)	−0.0028 (10)
C12	0.0469 (11)	0.0555 (13)	0.0485 (11)	−0.0001 (9)	0.0142 (9)	−0.0107 (9)
C11	0.0525 (12)	0.0471 (12)	0.0487 (11)	0.0010 (9)	0.0155 (9)	−0.0064 (9)
C10	0.0485 (11)	0.0472 (12)	0.0572 (12)	−0.0019 (9)	0.0197 (10)	−0.0134 (9)
C9	0.0594 (13)	0.0449 (12)	0.0653 (14)	−0.0011 (10)	0.0245 (11)	−0.0063 (10)
C8	0.0670 (14)	0.0505 (13)	0.0484 (12)	0.0005 (10)	0.0144 (10)	−0.0097 (9)
C7	0.0820 (15)	0.0581 (13)	0.0452 (11)	0.0039 (11)	0.0118 (10)	0.0004 (9)
O1	0.141 (2)	0.0451 (11)	0.0976 (15)	−0.0011 (11)	0.0388 (14)	−0.0120 (10)
C6	0.098 (2)	0.0475 (14)	0.0825 (19)	0.0032 (13)	0.0332 (17)	−0.0002 (13)
C5	0.0849 (15)	0.0702 (14)	0.0555 (11)	0.0096 (12)	0.0121 (11)	0.0049 (10)
C4	0.0867 (19)	0.0661 (16)	0.0595 (14)	0.0129 (13)	0.0186 (13)	−0.0165 (12)
C3	0.100 (2)	0.0675 (17)	0.0505 (13)	0.0057 (15)	0.0103 (13)	−0.0100 (12)
C2	0.0847 (19)	0.0693 (17)	0.0667 (16)	−0.0170 (14)	0.0193 (14)	−0.0210 (13)
C1	0.0772 (18)	0.097 (2)	0.0504 (14)	0.0074 (15)	0.0124 (13)	−0.0067 (14)

### Geometric parameters (Å, °)

**Table:**

S1—C1	1.683 (3)	C8—C2	1.541 (4)
S1—C12	1.716 (2)	C7—C5	1.432 (3)
O2—C10	1.352 (3)	C7—H7	0.867 (10)
O2—H2D	0.78 (3)	O1—C6	1.230 (3)
C15—C14	1.399 (3)	C6—H6A	0.99 (4)
C15—C10	1.402 (3)	C5—C1	1.339 (4)
C15—C12	1.476 (3)	C5—H5A	0.9500
C14—C11	1.391 (3)	C4—H4A	0.9800
C14—H14A	0.9500	C4—H4B	0.9800
C13—C11	1.374 (3)	C4—H4C	0.9800
C13—C9	1.396 (3)	C3—H3A	0.9800
C13—H13A	0.9500	C3—H3B	0.9800
C12—C7	1.408 (3)	C3—H3C	0.9800
C11—C8	1.544 (3)	C2—H2A	0.9800
C10—C9	1.403 (3)	C2—H2B	0.9800
C9—C6	1.457 (4)	C2—H2C	0.9800
C8—C4	1.527 (4)	C1—H2	0.902 (10)
C8—C3	1.530 (4)		
			
C1—S1—C12	92.60 (13)	C12—C7—H7	127 (3)
C10—O2—H2D	103 (2)	C5—C7—H7	122 (3)
C14—C15—C10	116.7 (2)	O1—C6—C9	125.0 (3)
C14—C15—C12	120.00 (19)	O1—C6—H6A	111 (2)
C10—C15—C12	123.30 (19)	C9—C6—H6A	124 (3)
C11—C14—C15	124.4 (2)	C1—C5—C7	113.4 (3)
C11—C14—H14A	117.8	C1—C5—H5A	123.3
C15—C14—H14A	117.8	C7—C5—H5A	123.3
C11—C13—C9	121.2 (2)	C8—C4—H4A	109.5
C11—C13—H13A	119.4	C8—C4—H4B	109.5
C9—C13—H13A	119.4	H4A—C4—H4B	109.5
C7—C12—C15	125.95 (19)	C8—C4—H4C	109.5
C7—C12—S1	110.58 (17)	H4A—C4—H4C	109.5
C15—C12—S1	123.46 (17)	H4B—C4—H4C	109.5
C13—C11—C14	117.3 (2)	C8—C3—H3A	109.5
C13—C11—C8	123.0 (2)	C8—C3—H3B	109.5
C14—C11—C8	119.69 (19)	H3A—C3—H3B	109.5
O2—C10—C15	118.8 (2)	C8—C3—H3C	109.5
O2—C10—C9	121.1 (2)	H3A—C3—H3C	109.5
C15—C10—C9	120.13 (19)	H3B—C3—H3C	109.5
C13—C9—C10	120.3 (2)	C8—C2—H2A	109.5
C13—C9—C6	119.3 (2)	C8—C2—H2B	109.5
C10—C9—C6	120.3 (2)	H2A—C2—H2B	109.5
C4—C8—C3	108.3 (2)	C8—C2—H2C	109.5
C4—C8—C2	109.5 (2)	H2A—C2—H2C	109.5
C3—C8—C2	108.3 (2)	H2B—C2—H2C	109.5
C4—C8—C11	109.58 (19)	C5—C1—S1	112.9 (2)
C3—C8—C11	111.9 (2)	C5—C1—H2	125 (2)
C2—C8—C11	109.16 (19)	S1—C1—H2	122 (2)
C12—C7—C5	110.5 (2)		

### Hydrogen-bond geometry (Å, °)

**Table:**

*D*—H···*A*	*D*—H	H···*A*	*D*···*A*	*D*—H···*A*
O2—H2D···O1	0.78 (3)	1.89 (3)	2.623 (3)	157 (3)
